# When More May Be Better: Reconceptualizing Clinical Handoffs in Modern Medicine

**DOI:** 10.7759/cureus.98443

**Published:** 2025-12-04

**Authors:** Kathryn E Berlin, Jian Zhang, Ke Yan, Susan Cohen, Sarah Yale

**Affiliations:** 1 Pediatrics/Neonatology, Medical College of Wisconsin, Milwaukee, USA; 2 Pediatrics/Quantitative Health Science, Medical College of Wisconsin, Milwaukee, USA; 3 Pediatrics/Hospital Medicine, Medical College of Wisconsin, Milwaukee, USA

**Keywords:** gastroschisis, handoff, safety patient, standardized handoff, length of stay

## Abstract

Background

Clinical handoffs are traditionally viewed as an area of vulnerability in patient safety, linked to miscommunication, adverse events, and prolonged hospitalizations. However, accreditation bodies have mandated standardized handoff training and processes, thereby embedding these practices into healthcare systems. In the neonatal intensive care unit (NICU), where complex and prolonged hospitalizations are common, handoffs may represent opportunities to enhance care. This study evaluated the relationship between the number of handoffs and length of stay (LOS) in infants with gastroschisis.

Methods

A retrospective cohort study was conducted of neonates with gastroschisis admitted to a level IV NICU between 2012 and 2020. Infants transferred before discharge or with incomplete records were excluded. Demographic, clinical, and surgical data were abstracted, including the presence of intestinal complications and closure type. Handoffs were quantified by the number of unique neonatologists and pediatric surgeons involved in care. Descriptive statistics and correlation analyses were performed, with multivariable models adjusting for severity and surgical covariates.

Results

Of 127 identified infants, 87 met the inclusion criteria. The median gestational age was 36 weeks, and 58% underwent primary closure. The median number of handoffs was 21, and the median length of stay (LOS) was 31 days. More handoffs per day were associated with shorter length of stay (r = -0.28, p = 0.0069). This association persisted after adjustment for confounders (β = -57.1, p = .034).

Conclusions

In this retrospective study, greater numbers of handoffs were associated with shorter hospitalizations in neonates with gastroschisis. Handoffs may provide opportunities for critical thinking, adaptive decision-making, and collaborative recognition of complications within complex sociotechnical systems. These findings highlight the need to reframe handoffs not solely as risks but as potential tools to optimize patient outcomes.

## Introduction

Handoffs have traditionally been viewed as a vulnerable area in terms of patient safety. Several studies have linked handoffs to an increased risk of miscommunications [1,2], adverse events [3], mortality [4], and extended hospital stays [5]. Handoffs are a necessary component of healthcare and have become more frequent due to changes in training duty hours and a growing understanding of the essential nature of provider wellness.

Accordingly, handoffs have been the focus of multiple accreditation bodies, including the Accreditation Council for Graduate Medical Education (which outlines standards for effective handoffs, including the need for formal training and assessment of competency [6]) and The Joint Commission (which requires all healthcare organizations to have standardized handoff processes [7]). These policies and changes have been in place for over 20 years and have become ingrained in the fabric of healthcare. It may be time to acknowledge that the risks associated with handoffs are no longer the same. Instead, we posit that the structured handoff of the modern era may be an opportunity to leverage the critical thinking and adaptive actions of providers who are part of a larger complex sociotechnical system (the actual work system that is made up of the interactions between people, tools, tasks, and environment) [8,9].

In the neonatal intensive care unit (NICU), complex patients are commonplace, with one known population being babies born with gastroschisis. Gastroschisis is a condition in which a congenital ventral wall defect prevents the bowel from returning into the abdomen during development, resulting in eviscerated bowel at birth. Clinical care includes abdominal closure, which can be either primary (immediately after birth) or secondary. Patients with gastroschisis may have prolonged hospitalizations in the setting of surgical recovery, a delay in the return of bowel function, and feeding intolerance. During these prolonged hospitalizations, numerous handoffs occur between providers. As such, we sought to evaluate the relationship between the frequency of clinical handoffs and the duration of hospitalization to assess the impact of handoffs on patient care.

## Materials and methods

This was a retrospective cohort study of all neonates with gastroschisis admitted to and discharged from a single-center level IV NICU at Children’s Wisconsin (Milwaukee, WI) from January 1, 2012, to December 31, 2020. We excluded infants for whom the diagnosis of gastroschisis versus omphalocele was unclear. We also excluded those with incomplete hospital records (generally secondary to a transfer of care from our institution to another hospital system) or those who died before discharge, as this would artificially shorten the length of stay.

Patient and family demographics, as well as NICU illness covariates, were collected via chart review. These included gestational age, small for gestational age, birth weight, birth location (inborn versus born at an outside facility), infant sex, delivery mode, duration of mechanical ventilation, comorbidities, and the presence of intestinal necrosis, matting, atresia, or perforations on admission. Surgery covariates, including type of closure (primary versus secondary), age at definitive closure, and need for general anesthesia, were also collected. Outcome variables were obtained via chart review, and the covariates included enteral feeding data and discharge variables such as weight at discharge, feeding plan at discharge, and length of stay (LOS). To quantify handoffs, the total number of different providers (neonatologists and pediatric surgeons) a patient had during their NICU stay, and the number of handoffs between them were tracked. The handoff to the on-call team at night was omitted, as it would be directly proportional to the LOS. This study was approved by the Children’s Wisconsin Institutional Review Board (IRB) (Approval No. 1727281-1). 

Descriptive statistics, including mean, standard deviation, median, quartiles, minimum, and maximum, were summarized for continuous variables. Frequency count and percent were generated for categorical variables. Pearson’s and Spearman’s correlation coefficients were calculated for the number of handoffs per day and LOS. A general linear model (GLM) was used to evaluate the association between these two variables after adjusting for covariates, which include the presence of intestinal matting, the silo placement, and having any complications. All covariates were complete with no missing values. A p-value <0.05 was considered statistically significant. The statistical software SAS 9.4 (SAS Inc., Cary, NC, USA) and IBM SPSS Statistics for Windows, version 29 (IBM Corp., Armonk, NY, USA) were used for the analyses.

## Results

In total, 127 infants with gastroschisis were identified during the study period, with 87 included in the final analysis. Four were excluded secondary to death, and an additional 36 were excluded secondary to incomplete records, generally secondary to transfer of care to a different hospital system. Patient demographics are shown in Table 1. Median gestational age at birth was 36 weeks (IQR 35-36); 33% had some intestinal matting or necrosis noted on admission. Primary fascial closure was performed in 62% of cases. Complications after closure occurred in 39% of patients.

**Table 1:** Demographics Descriptive statistics (numbers, percentages, medians, and interquartile ranges as appropriate) were used to summarize demographic characteristics.

Demographics (n=87)		
Male sex (n, %)		48 (55%)
Gestational age (n, %)	31 to 33 weeks	9 (10%)
	34 to 36 weeks	73 (84%)
	37 to 39 weeks	5 (6%)
Delivery mode (n, %)	Vaginal	65 (75%)
	Cesarean section	22 (25%)
Birthweight (kg) (Median (IQR))		2.33 (2.10-2.65)
Multiple gestation (n, %)		2 (2%)
Outborn (n, %)		7 (8%)
Closure type (n, %)	Primary	54 (62%)
	Delayed	33 (38%)
Intestinal matting (n, %)	None	59 (68%)
	Mild	15 (17%)
	Severe	13 (15%)
Silo placed (n, %)		32 (37%)
Complications (n, %)		13 (15%)
Readmitted within one year of discharge (n, %)		42 (33%)

**Figure 1:**
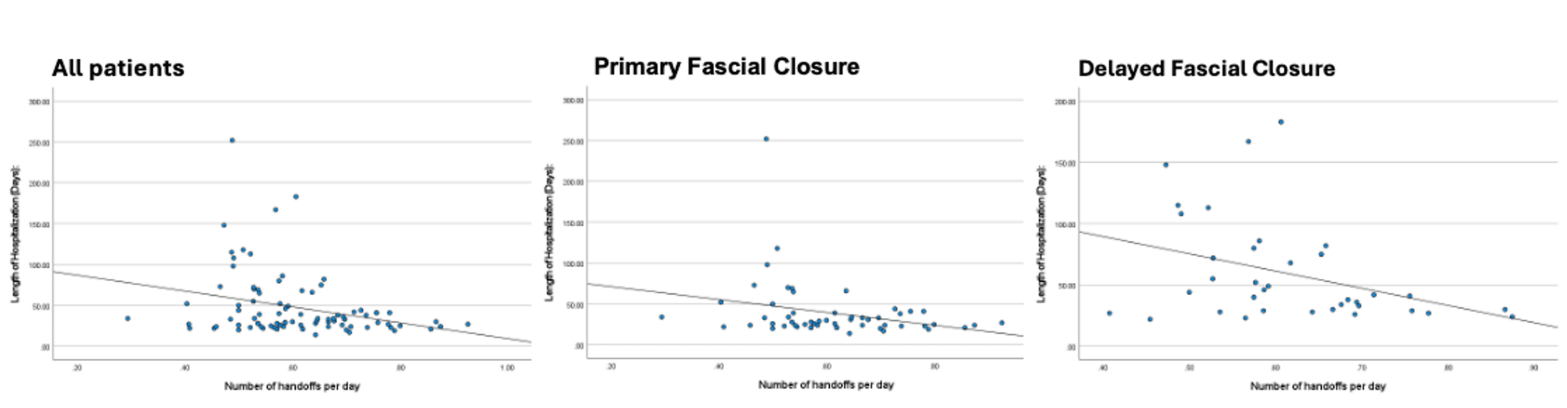
Correlation between number of handoffs and length of stay An increased number of handoffs was associated with shorter length of stay. This negative correlation was observed overall (r = -0.29, p = 0.0047) and when stratified by closure type (primary closure with r = -0.28, p = 0.04 and secondary closure with r = -0.37, p = 0.029).

The median number of handoffs during NICU admission was 21 (IQR 15-30). The median length of stay for these patients was 31 days (IQR 24-49), with a median postmenstrual age at discharge of 40 weeks (IQR 39-42). With more handoffs, the trend demonstrated a shorter length of stay (r = -0.28, p = 0.0069). This trend persisted even when patients were divided into groups based on type of closure (primary closure: r = -0.28, p = 0.041; secondary closure: r = -0.35, p = 0.045). Figure 1 demonstrates these correlations. 

After adjusting for the presence of intestinal matting, silo placement, and complications with a general linear model, the increased number of handoffs per day remained significantly associated with shorter LOS (β = -57.1, p = 0.034), as shown in Table 2.

**Table 2:** Multivariable model adjusting for covariates This table summarizes the results of a general linear model evaluating the association between the number of daily handoffs and the outcome, adjusted for intestinal matting, silo placement, and complications.

Independent variable	Coefficient (SE)	p-value
Number of handoffs per day	-57.1 (26.5)	0.034
Intestinal matting		
None (reference)	—	—
Mild	16.2 (8.7)	0.066
Severe	9.5 (10.0)	0.35
Silo placed		
No (reference)	—	—
Yes	9.3 (7.3)	0.21
Complication		
No (reference)	—	—
Yes	51.7 (8.5)	<0.0001

## Discussion

In this study of neonates with gastroschisis, LOS was negatively associated with the number of clinical handoffs, and this association persisted even after adjustment for severity variables. While handoffs have traditionally been viewed as a risk to patient safety, it may be time to reconsider this perspective. First, over the past twenty years, significant efforts have been made to improve the handoff process [10]. Previous studies have shown decreases in medical errors following the implementation of structured handoff programs [11] and in preventable adverse events [12].

Handoffs also provide the opportunity to re-evaluate diagnostic schemas and assess the patient with fresh eyes, improving outcomes by catching previously missed information. A 2013 study noted that the odds of mortality in the adult ICU decreased by 23% for each additional day of nighttime cross-coverage in the first seven days of ICU admission [13]. One review likened this to a “second opinion” nightly [14]. This parallels the findings of our analysis and suggests that a fresh perspective may be key to improving care.

The novel finding in this study was that the length of hospital stay was negatively associated with the number of clinical handoffs. This association remained statistically significant in the whole cohort after adjustment for severity variables. We propose that providers use clinical handoffs as an opportunity to actively engage in team dialogue, critical thinking, and adaptive actions within the sociotechnical system, thereby leading to more timely and accurate diagnoses of patient complications. A human factors systems approach is crucial to improving patient safety [15], and this sociotechnical lens should be applied to handoffs as well. Prior work has evaluated outcomes related to specific components of the sociotechnical system, such as how electronic tools can support provider handoff [16]. With increasing incorporation of human factors principles, this work can be expanded to design systems (including handoffs) that improve the performance of providers and reduce hazards [17]. As a starting point, we can shift our perspective on clinical handoffs and recognize them as opportunities to leverage a crucial aspect of our complex system: the expertise and critical thinking of providers on the front lines.

While this study demonstrates that an increased number of handoffs may reduce hospital stay length for this population, we acknowledge several limitations. These factors include varying levels of disease severity and comorbidity among the patient cohort, the inability to access the content of handoff material through retrospective chart review, and the challenge of determining direct causality, as other undefined variables may have influenced the relationship between the number of handoffs and the length of hospital stay. Furthermore, our decision to exclude patients transferred to other systems or with incomplete records to avoid skewing LOS may have biased the results by removing patients who were not complex enough to require Level IV care.

Despite these limitations, this data may provide a foundation for a new perspective, one in which an increased number of clinical handoffs in the NICU is not always a negative. Although gastroschisis involves a small patient population, this cohort demonstrates that, within a complex sociotechnical system, handoffs can improve clinical outcomes and reduce hospital LOS in this population. Future work should further explore these findings with other patient populations, as this may be generalizable to other complex populations, including those outside of the NICU, and use a sociotechnical lens to optimize medical decision-making and enhance patient care outcomes.

## Conclusions

While handoffs have been historically perceived as having an adverse impact on patient safety, this retrospective study suggests that they may play a beneficial role in neonates with gastroschisis. In a single-center NICU, a greater number of handoffs was associated with a shorter LOS, even after adjusting for clinical severity and complications. These findings suggest that handoffs serve as an opportunity for critical thinking and collaborative problem-solving, challenging our traditional assumption that they are associated with increased risk. Future research should examine this conclusion in other populations to determine if this association persists.
